# Machine learning methods for estimating bent photonic crystal fiber based SPR sensor properties

**DOI:** 10.1016/j.heliyon.2022.e11582

**Published:** 2022-11-10

**Authors:** Cem Kalyoncu, Ahmet Yasli, Huseyin Ademgil

**Affiliations:** a European University of Lefke, Faculty of Engineering, Computer Engineering Department, Lefke, TR-10, Turkey; b European University of Lefke, Faculty of Engineering, Electrical and Electronics Engineering Department, Lefke, TR-10, Turkey

**Keywords:** k-nearest neighbor regression, Machine learning, Photonic crystal fiber sensor, Surface plasmon resonance, Optical sensing

## Abstract

In the recent years, the use of machine learning approaches in optical devices and fibers is increasing. However, most methods concentrate on the use of Artificial Neural Network (ANN) methods due to the ability of automatically fitting to the problem. In this work, a classical non-linear regression method, namely k-Nearest Neighbor Regression (KNNR) is proposed for determining the loss characteristics of a photonic crystal fiber (PCF) based surface plasmon resonance (SPR) sensor in the presence of a bend in either *x* or *y* direction. Although KNNR is a simple method, it is very well known that in certain systems it can out-perform ANN. It is believed that PCF based structures can be a good candidate for this comparison. In order to judge the performance of different regression techniques, we have built a database that contains 1180 samples. The dataset contains PCF structure data for non-bent(straight fiber), bent in *x* and *y*-directions. Experiments show that KNNR outperforms both ANN and Linear Least Square Regression methods even when a feature space expansion method is employed. In addition, KNNR does not require any lengthy training process, allowing it to be used instantly once the training data is available. This can be exploited to complement existing simulation techniques.

## 1 Introduction

In the recent years, optimizing and refinement of microstructured optical devices with considerable low computational time is becoming crucial. For this purpose, genetic algorithms, artificial neural networks (ANN), and various other machine learning (ML) algorithms [1], [2], [3], [4], [5], [6], [7], [8] were employed in the past. In this study, ML approaches are proposed to estimate the bending loss characteristics of a PCF based SPR structure. The comparisons are performed between two conventional techniques, namely, Linear Least Squares Regression (LLSR) [9], k-Nearest Neighbor Regression (KNNR); and a commonly used Artificial Neural Networks (ANN) technique that has been used in multiple similar studies [2], [3], [4], [5]. A feature space expansion method is also employed to improve the scores of LLSR and ANN systems.

Initially, PCFs were designed and developed for communication applications [10]. However, in the last decade, these extraordinary structures have begun to be designed for a wide range of sensing applications [11]. The design flexibility and possibility of adaptation to various environments make these structures indispensable, especially in biomedical and chemical applications [12], [13], [14]. Surface Plasmon Resonance is a prevalent optical sensing technique that became an ideal candidate for real time, label free, and accurate remote sensing purposes. These distinctive structures are designed with traditional prism-coupled configuration [12], [15]. In addition to having a larger dimensions compared to other optical sensing solutions, the need for additional mechanical components makes these structures far from being an ideal sensor model. In this context, the desired propagation characteristics for sensing applications can be achieved by using PCF and SPR technologies together. Recent studies [16], [17] have shown that PCF based SPR sensors are promising high sensitivity levels for various analytes. This technological combination makes it possible to design highly sensitive multi-channel [15], [17], multi-analyte [18], bending insensitive structures [19], which can be employed for temperature, humidity, gaseous and liquid sensing purposes [14], [16], [17], [20]. In PCF based sensors, several types of metallic layers can be employed to inspire SPR. Gold (Au) and silver (Ag) appeared as the most commonly used plasmonic layers. Recently various metallic layers such as Ta_2_O_5_ [15], ITO [13], TiO_2_ [12] have also attracted the attention of researchers.

The performance of PCF based structures is mainly analyzed with numerical methods [12], [15], [16], [18], [21]. These methods are beam propagation, effective index, plane wave expansion, localized function expansion, multipole, finite difference, Fourier decomposition and finite element method (FEM). Each method exhibits some advantages and disadvantages. The most critical metric is the accuracy of the numerical method, where FEM emerges as the most reliable method for PCF based structures. Additionally, FEM becomes more attractive as it allows the full exploitation of potential structural symmetry to reduce the physical computational domain and the simulation time [10].

In general, ML techniques can be employed on optics and photonics systems to reduce the complexity of designed structure and thereby reducing the computation time. Furthermore, ML can be used to map the critical design parameters such as cladding geometry and material. Finally, prediction of critical propagation characteristics such as confinement loss, dispersion, birefringence and sensitivity can be geared up with appropriate ML modeling [1], [5], [14].

Previous investigations have shown that bending is very critical on the overall performances of SPR based sensor devices [22], [23]. It has been reported that bending has a positive impact on the evanescent wave intensity [22] of fiber based SPR biosensors. Similarly, Su et al. [23] have experimentally considered the effects of bending on the measurement of curvature and temperature. Therefore, analyzing and predicting the bending behavior of such sensor structure becomes crucial for various sensing applications. Authors believe that predicting the optimized propagation characteristics and the effects of bending faster than numerical methods is just possible with ML modeling.

In this paper, we propose KNNR to estimate the loss of a PCF based SPR structure in the presence of bend. KNNR is a non-linear regression system that can estimate the result of a system effectively. In the past, conventional ANN [2], [3], [4], [8] and generative adversarial network (GAN) [5] models have been employed for PCF based structures. These methods need substantial training time. However, KNNR does not require a training phase and can be used as soon as the training data is ready. Furthermore, new data can be added to the training data without any additional calculation. To the best of our knowledge, there are no published work that investigates such structures with KNNR method. Furthermore, this study contains the comparison of these specific ML models.

The rest of the paper is organized as follows: in Section 2, the design parameters of the proposed PCF based SPR sensor and the details of produced dataset that are used in the experiments are discussed. Section 3 contains the methods that are used in the experiments. The experimental results that highlights the effectiveness of KNNR model are presented in Section 4. Finally, concluding remarks and future works are presented in Section 5.

## 2 PCF based SPR sensor design and dataset

**Figure 1:**
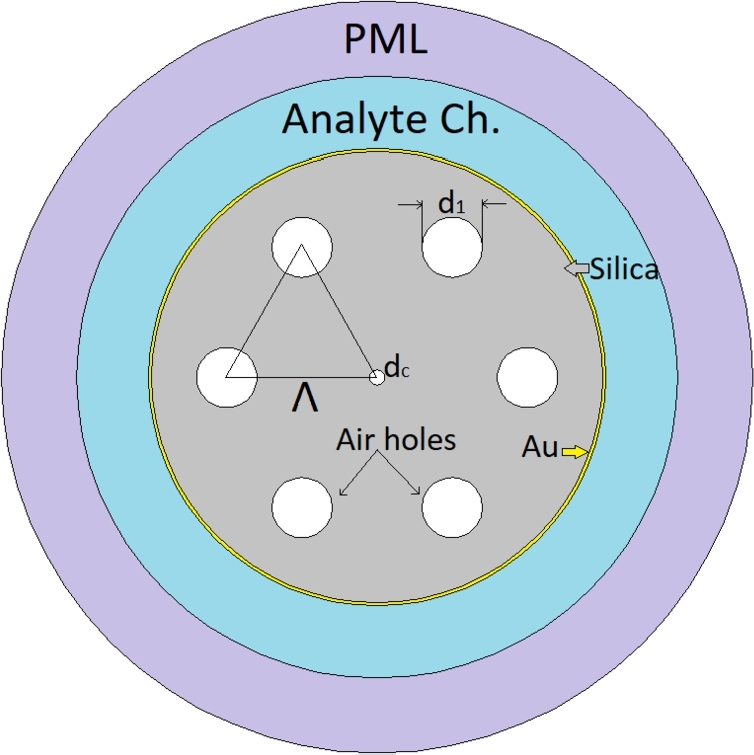
The cross section of proposed PCF based SPR sensor.

In Fig. 1, the cross-section of the sensor structure that is used to build the desired dataset is illustrated. The sensor structure consists of an analyte channel (detection medium), plasmonic layer, and photonic crystal fiber. As a plasmonic layer, silver (Ag) provides sharper resonance peaks compared to gold layer, however, practically additional metallic layer such as graphene is needed to protect silver layer from oxidation. This may complicate the fabrication procedure, therefore, in this study, gold (Au) layer is employed as plasmonic material. The PCF section consists of seven various sized air holes placed on a silica background with a hexagonal lattice arrangement. The air hole diameter on the first layer is $d_{1}=0.8\;\mu \text{m}$ and the central air hole is $d_{c}=0.2\;\mu \text{m}$. The hole to hole distance (Λ) is set to 2μm. Gold (Au) material is used as a plasmonic layer and is set at 40 nm. Johnson and Christy Data [24] were used for permeability values. The well known Sellmeier Equation is used to calculate the refractive index of the silica material. As delineated in Eq. (1), *λ* is the operating wavelength, *n* is the Refractive Index (RI), $B_{1,2,3}$ and $C_{1,2,3}$ are the Sellmeier constants [10].

**Figure 2:**
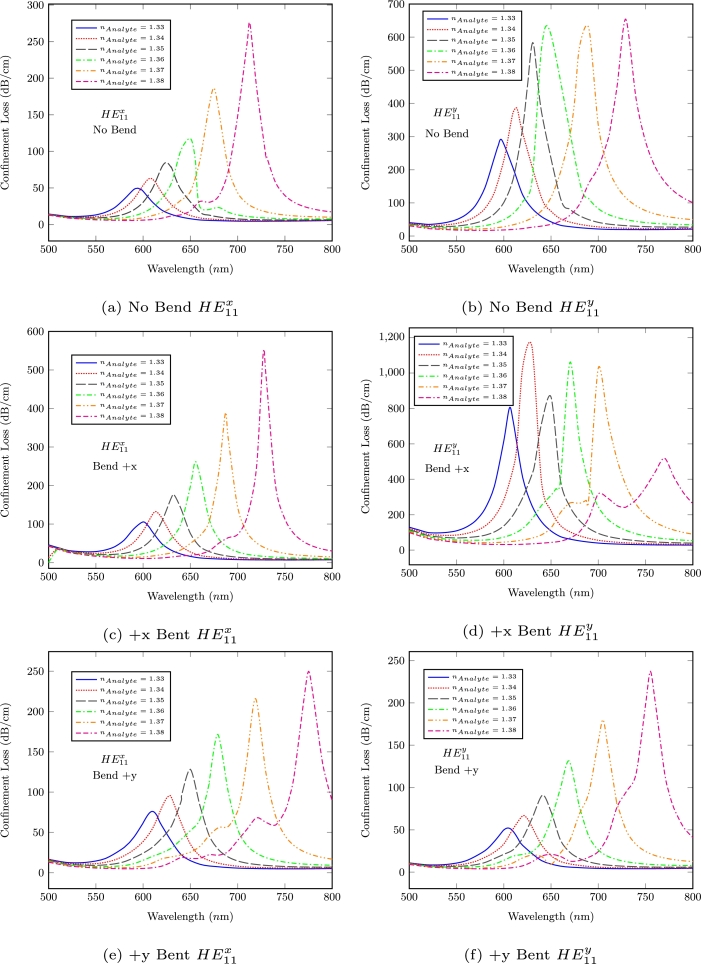
The confinement loss characteristics of proposed sensor for all bend conditions.

The confinement loss characteristics of the proposed sensor for straight and bent conditions have been presented in Fig. 2. Fig. 2 a and b illustrates the loss characteristic of fundamental $HE_{11}^{x}$ and $HE_{11}^{y}$ modes, respectively. In Fig. 2 c and d, the confinement losses of fundamental $HE_{11}^{x}$ and $HE_{11}^{y}$ modes are presented where the +x direction bent applied by using Eq. (3). Similarly, Fig. 2 e and f shows the loss characteristic with +y direction bent. It can clearly be seen from all figures that; the loss peaks are shifted towards longer wavelengths with increase in analyte RI. As it is well known, variation at peak wavelengths is used for the spectral sensitivity [25] analysis as shown in Table 1.

(1) $$n(\lambda )=\sqrt{1+\frac{B_{1}{\left(\lambda \right)}^{2}}{{\left(\lambda \right)}^{2}-C_{1}}+\frac{B_{2}{\left(\lambda \right)}^{2}}{{\left(\lambda \right)}^{2}-C_{2}}+\frac{B_{3}{\left(\lambda \right)}^{2}}{{\left(\lambda \right)}^{2}-C_{3}}}$$

**Table 1:** The sensitivity analysis of proposed PCF-SPR sensor for all variations of RI changes.

Bend	RI	Spectral Sensitivity		Resolution	
	Range	(nm/RIU)		(RIU x $10^{-5}$)	
		$HE_{11}^{x}$	$HE_{11}^{y}$	$HE_{11}^{x}$	$HE_{11}^{y}$
No	1.33-1.34	1400	1500	7.1	6.7
	1.34-1.35	1700	1800	5.9	5.6
	1.35-1.36	2500	3000	4	3.3
	1.36 -1.37	2500	2900	4	3.4
	1.37-1.38	3800	3900	2.6	2.5
+x	1.33-1.34	1400	2400	7.1	4.2
	1.34-1.35	1900	2000	5.3	5
	1.35-1.36	2200	2000	4.54	5
	1.36 -1.37	3200	3000	3.1	3.3
	1.37-1.38	4000	7000	2.5	1.4
+y	1.33-1.34	1800	1600	5.6	6.3
	1.34-1.35	2200	2100	4.5	4.8
	1.35-1.36	2900	2800	3.4	3.5
	1.36 -1.37	4000	3600	2.5	2.7
	1.37-1.38	5600	5000	1.7	2

The confinement losses reach their highest rates (peak point) when the resonance phase matching occurs. Therefore, it is critical to precisely calculate the confinement losses. The well known equation is shown in Eq. (2), where the imaginary part of the effective RI shown with Im($n_{eff}$).

(2) $$\frac{40\pi }{ln(10)\lambda }Im(n_{eff})\times 10^{4}[\text{dB/cm}]$$

A labeled dataset of 1180 samples was provided by the numerical analysis of full vectorial FEM method. The confinement loss was precisely calculated for six different analytes (n=1.33, 1.34, 1.35, 1.36, 1.37, 1.38). In FEM analysis, the accuracy of the results is ensured with finer meshing. The sensor structure was divided into 11298 triangular subspaces.

Bending analysis of the proposed structure is calculated with the following equation [19]:

(3) $$n_{eq}=n(x,y)exp\left(\frac{X}{R}\right)$$

where n(x, y) and R denotes the RI profile of the straight fiber and bend radius.

**Figure 3:**
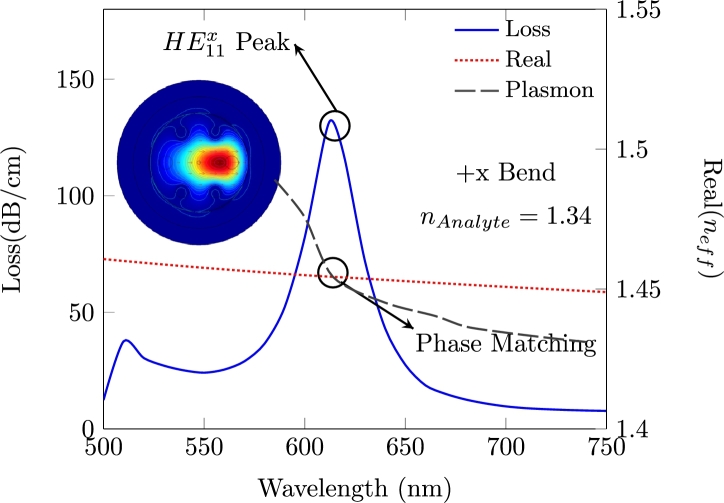
The resonance condition of proposed PCF-SPR sensor with +x bend.

**Figure 4:**
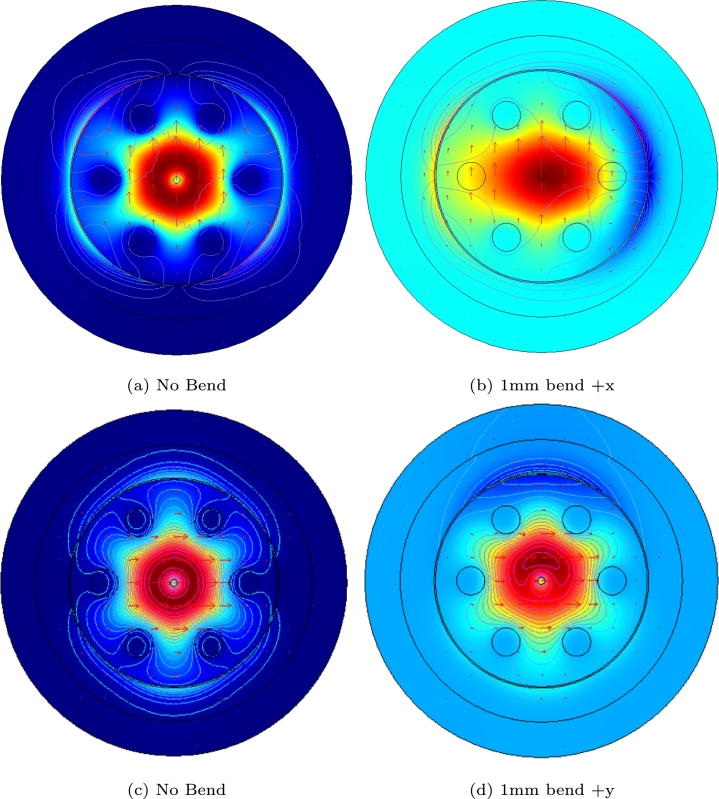
The magnetic field distribution for no bend, +x and +y bend conditions.

In Fig. 3, the real part of effective refractive indices of both plasmon-x and fundamental modes of +x bent structure are presented. The analyte channel is filled with 1.34 refractive index. It is clearly seen that the confinement losses are reaching to their peak (highest) level at 614 nm wavelength. This phenomenon is called phase matching, where plasmon-x and fundamental modes are intersecting. Meanwhile the magnetic field distribution of resonance matching condition of bend and straight structure is illustrated in Fig. 4. As can be seen from the figures, the fundamental modes are slightly shifted to towards the bending direction of the structure.

The sensor performance for bent and straight conditions is summarized in Table 1. The highest spectral sensitivities for straight structure (3900 nm/RIU and 3800 nm/RIU) are obtained at 1.37 and 1.38 RI range. On the other hand, the sensitivity values of +x direction bent structure have increased to 4000 nm/RIU and 7000 nm/RIU for same RI range. Similarly, sensitivity levels are increase up to 5600 nm/RIU and 5000 nm/RIU +y direction bent structure.

## 3 Methodology

In this work, different regression systems have been examined in order to obtain the loss characteristics of a sensor structure. These experiments also include the bending analysis of structure in a cardinal direction. This study focuses on three commonly used regression methods, namely KNNR, LLSR, and ANN, with and without the feature space expansion technique that is discussed in the last subsection.

### 3.1 KNNR

KNNR is a classical non-linear regression system where the output of a sample is determined by the closest samples that are known. Although this method is commonly used in many fields, to our knowledge, it has not been explored to be used in optical structures. KNNR is closely related to KNN classifier, however, unlike classifiers, regression methods must produce a value rather than a label.

The working principle of KNNR is to find K samples that are closest to the unknown sample. Different distance metrics can be used to determine which samples are closest. The experiments are carried out with Euclidean and Manhattan distance metrics. After the neighboring samples are determined, their values are reduced to a single value and used as the output of the unknown sample. There are different techniques that can be applied at this step. The most common method is to use the inverse distance as the weight. We generalize this metric with a parameter that allows us to modify the falloff. This equation is described in Eq (4), where *S* is the parameters of the unknown sample, *C* is the parameters and *O* is the outputs of closest k samples, **D** is the distance function, and *σ* is the parameter of this system.

(4) $$output=\sum_{i=1}^{k}\frac{O_{i}}{\text{D}{\left(S-C_{i}\right)}^{\sigma }}$$

Previous studies [26], [27] have found higher powers of Euclidean distance capture the natural data distribution better than the Euclidean distance itself. This notion is also supported by the experiments in Section 4.

### 3.2 Linear least squares regression

LLSR is a common regression technique that is known to work on linear or log-linear data. In our experiments LLSR is employed to form a baseline. Although it is a simple method, LLSR requires matrix inversion for training; therefore, its training time is relatively long. This is documented in Section 4. ANNs can easily emulate LLSR via training, however, in most cases, LLSR is a faster regression system after the training phase is completed.

LLSR requires parameter vector *β* to be trained using Eq. (5) where *X* is the matrix of training samples and *y* is the vector of desired outputs. If the number of parameters are high, matrix *X* could be singular. Once the training is completed, estimated output of a given sample can be calculated using Eq. (6) where *N* is the number of parameters and *x* is the input sample. In LLSR, an additional input parameter of 1 is often added to every sample, allowing system to have offsets. For uniformity and to assist with feature expansion, 1 is added as an additional parameter in all regression methods used in this research.

(5) $$\overset{ˆ}{\beta }={\left(X^{⊺}X\right)}^{-1}X^{⊺}y$$

(6) $$O=\sum_{i}^{N}\beta_{i}x_{i}$$

Although LLSR is used in many fields as the primary regression system, its success depends on the underlying system. In chaotic systems such as PCF structures the success of LLSR cannot be guaranteed.

### 3.3 Artificial neural networks

ANN is the most commonly used regression system in photonics structures [2], [3], [4], [5], [6], [7], [8]. It is a generalized system that can be used on any type of classification or regression task. The popularity of ANN comes from the fact that it can be deployed with minimal modifications to its design and without the need for any parameter preprocessing. However, it is possible to improve ANN accuracy and potentially reduce training time with parameter preprocessing.

In our proposed model, a feed-forward neural network with back-propagation is employed to analyze the effectiveness of ANN systems. In order to find the optimal ANN system for this task, two sets of experiments are performed. The first set is to analyze and find a good starting point for the ANN architecture. The second set is designed to optimize the hyper-parameters of the designed ANN architecture. According to these experiments, we have found using two hidden layers with Rectified Linear Unit (ReLU) activation function results in the minimal testing error when batch normalization is applied on the input. The second experiment helped us to determine hidden layer size, batch size and the number of epochs for training. Details of this optimal ANN design are given in Table 2. Details from this experiment are available in Section 4.

**Table 2:** Details of ANN model.

Parameter	Regressor
Hidden layers	2
Neurons in hidden layers	200
Batch size	128
Activation function	ReLU
Optimizer	Adam [28]
Input normalization	Batch [29]
Loss function	Mean absolute percentage error

### 3.4 Feature space expansion using higher order features

Due to the fact that the number of parameters are low in the proposed system, it could be beneficial to expand the feature space. In this work, feature space is expanded by using higher order features obtained by multiplication. Since the first parameter is set to 1, simply multiplying each independent parameter with each other will yield first and second order features. When this operation is repeated, the result will produce first, second, third and fourth order features. Eq. (7) shows this operation is mathematical form where I2 is the expanded input and *I* is the original input.

(7) I2={ij|i∈I,j∈I}

Generally, this operation is necessary in linear regression systems such as LLSR. However, experiments show that this also increases the accuracy of ANN. Although ANN systems can build multiplicative relations within their hidden layers, such relations require more training data and training epochs to form. When the underlying system contains such multiplicative relations directly, adding them will allow us the shrink the size and the depth of the hidden layers as well reducing the training epochs. Additionally, decreased training epochs will reduce the impact of memorization when the number of samples are not enough for the training.

KNNR uses input parameters only for distance calculation. Since higher order features are calculated multiplicatively, they do not change distances in any meaningful way. Therefore, accuracy of KNNR is not affected by this feature space expansion method. However, increased number of features increases computational demand of the operation.

The effect of feature space expansion is analyzed in Section 4.

## 4 Experimental results and discussions

In order prove the effectiveness of KNNR for bent PCF based SPR sensors. Extensive experiments have been performed, comparing KNNR model with LLSR and ANN models. All experiments are performed on AMD 3700X Linux PC with 32 Gb of RAM. Classical methods are programmed in C++, whereas TensorFlow with PyTorch is used for ANN. Since the number of features are too few, GPU acceleration does not improve the speed of the computation. 10-fold testing used to obtain the results. In this work, we have opted to use Mean Absolute Percentage Error (MAPE) metric as the output range varies depending on the sample. Eq. (8) describes the formula for MAPE metric.

(8) $$MAPE=100\left|\frac{target-output}{target}\right|$$

**Figure 5:**
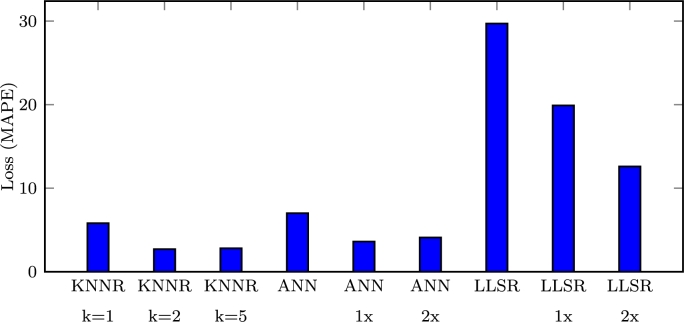
Loss of different regression methods. 1x and 2x refers to feature space expansion.

The first experiment involves overall deviation from the ground truth in percentage. The critical findings of this experiment are shown in Fig. 5 where the effect of different *k* values for KNNR and different feature space expansion applications for ANN and LLSR methods is listed. 1x and 2x shown in the figure refers to feature space expansion. 1x expansion includes second order features while 2x includes second, third, and fourth order features. For KNNR, *σ* is set to 4 and we have used the hyper-parameters that results in the minimal loss for ANN. The feature space expansion for KNNR has not been included as it does not have any effect on the loss. This experiment clearly demonstrates that KNNR with k≥2 has lower loss levels compared to both ANN and LLSR methods.

**Figure 6:**
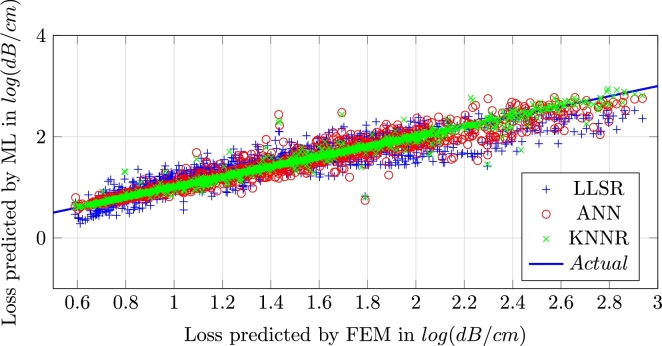
Testing results of all samples.

Comparison of regression methods for all samples in the database is shown in Fig. 6. This graph is also produced by 10-fold testing. For LLSR, feature space expansion is used twice, while it is used once for ANN method.

Even when the parameters are not finely tuned, KNN regression still produces acceptable results. Using higher *σ* value for the distance factor reduces the chances of larger *k* values from lowering the accuracy; while increasing k value actually reduces the chance of results getting affected by noise, even though overall loss is slightly increased. The parameter analysis of KNNR is presented in Table 3. Even though experiments are performed with both Euclidean and Manhattan distance metrics, Manhattan distance metric results were excluded as the loss of both metrics are within the expected deviation of the experiments. Therefore, we can argue that both distance metrics can be used for this task.

**Table 3:** KNNR loss values in MAPE using Euclidean distance metric.

*σ* →	0	1	2	3	4	5	8
k ↓							
1	5.8%	5.8%	5.9%	5.8%	5.8%	5.8%	5.8%
2	3.0%	**2.7%**	**2.7%**	**2.7%**	**2.7%**	2.8%	2.8%
3	4.6%	3.6%	3.0%	2.9%	2.8%	2.8%	2.8%
5	5.8%	4.2%	3.3%	3.0%	2.8%	2.8%	2.8%
8	8.3%	5.5%	3.7%	3.1%	2.9%	2.8%	2.8%
11	10.4%	6.5%	4.1%	3.2%	2.9%	2.9%	2.8%
15	10.7%	7.1%	4.3%	3.2%	2.9%	2.8%	2.8%

The feature space expansion and its effects on the results are tested in a separate experiment. The detailed explanation of the conducted method is included in Section 3. Experiments indicate that both in LLSR and ANN, using higher order features reduces the loss. However, adding higher order features has a tipping point. According to our experiments, KNNR is not affected by higher order features, ANN only benefits from second order features and finally LLSR can benefit up to and including fourth order features. Adding higher order features beyond these numbers increases the training and the testing time as well as the average loss of the overall system.

In this work, many alternative configurations and hyper-parameters for the ANN regression system have also been explored. This includes using more and less hidden layers (from 1 to 5 layers are tested), using different activation functions, applying batch normalization and dropout to hidden layers. It is found that the listed configuration in Section 3 has the best results among these trials. Additionally, we have tested adding higher order features, likewise, it does not improve the overall result even with higher number of epochs and network size. Some of the results from this experiment is demonstrated in Table 4.

**Table 4:** Hyper-parameter analysis of ANN regression, size is the number of nodes in hidden layers.

No expansion																	
Size	→	25				50				100				200			
Batch	→	32	64	128	256	32	64	128	256	32	64	128	256	32	64	128	256
Epochs	↓																

	100	11.9%	9.5%	9.3%	11.2%	12.0%	9.3%	9.0%	10.0%	11.6%	8.8%	8.6%	8.8%	11.0%	9.0%	8.7%	8.7%
	200	11.4%	9.0%	8.0%	8.9%	11.5%	9.0%	8.0%	8.5%	11.0%	8.5%	8.1%	8.0%	10.7%	8.0%	8.2%	8.6%
	300	11.4%	8.6%	7.8%	8.4%	11.0%	8.1%	7.7%	7.6%	11.0%	8.5%	7.7%	7.7%	10.9%	8.4%	**7.0%**	7.7%
	400	11.1%	8.3%	7.7%	8.2%	11.2%	8.5%	7.4%	8.1%	10.8%	8.1%	7.5%	7.4%	11.0%	8.1%	7.3%	8.0%
	Avg	11.7%	9.3%	9.1%	10.5%	11.6%	9.0%	8.5%	9.5%	11.3%	8.7%	8.2%	8.6%	11.2%	8.5%	8.1%	8.8%
	Avg	10.17%				9.66%				9.20%				9.14%			

1x Expansion																	

Size	→	100				200				300				500			
Batch	→	32	64	128	256	32	64	128	256	32	64	128	256	32	64	128	256
Epochs	↓																

	200	8.9%	5.3%	5.2%	5.8%	8.7%	5.0%	4.8%	6.3%	8.7%	5.4%	5.0%	5.7%	8.8%	4.8%	4.7%	5.2%
	400	8.6%	4.6%	4.1%	4.5%	8.1%	4.6%	4.5%	4.2%	8.6%	4.5%	3.8%	4.6%	8.3%	4.9%	3.9%	4.8%
	600	8.3%	4.4%	4.1%	4.0%	8.4%	4.4%	**3.6%**	3.8%	8.1%	4.1%	**3.6%**	3.8%	8.1%	4.5%	4.0%	3.6%
	800	8.0%	5.4%	4.3%	4.5%	7.8%	5.2%	4.3%	4.6%	8.0%	5.0%	3.9%	4.4%	7.9%	5.1%	3.8%	4.2%
	1000	7.7%	5.1%	4.1%	4.5%	8.0%	4.8%	4.1%	4.0%	7.8%	5.0%	4.1%	3.7%	8.0%	4.9%	3.9%	4.3%
	Avg	8.6%	5.5%	5.1%	5.7%	8.5%	5.3%	5.0%	5.6%	8.6%	5.3%	4.9%	5.4%	8.7%	5.3%	4.8%	5.4%
	Avg	6.23%				6.11%				6.04%				6.04%			

The final experiment is performed to illustrate and compare the computational speed of the methods. The training and testing times are presented in Table 5. In many systems, testing time is the most critical aspect and according to this table, LLSR has the fastest speed and KNNR is the slowest. Even though ANN is far faster than KNNR in testing time, 270000 tests are required for ANN to makeup for the training time. Additionally, it is possible to speed up KNNR using multiple treads. However, it should be noted that the testing time of KNNR is directly proportional to the training data size and can increase or decrease depending on the number of samples available.

**Table 5:** Computational speed comparison in milliseconds.

Method	Training	Testing
LLSR	0	0.02
LLSR 1x	13	0.02
LLSR 2x	10563	0.02
ANN⁎	10177	0.03
ANN⁎ 1x	18886	0.04
KNNR	0	0.11
FEM	-	9026

⁎ ANN uses multi-threaded computation

## 5 Conclusion

In this study, benefits of employing classical regression techniques in PCF based sensor models are discussed. Additionally, a detailed comparison of these methods is presented. Specifically, the experiments highlight the strength of KNNR in PCF based structure's loss estimation in the presence of bends. A prominent benefit of KNNR is its robustness towards its hyper-parameters. Once the *σ* is set to 6 and above, higher *k* values do not have negative effect on the outcome. Higher *k* values increase the number of samples participate in the output, and if exist, reduce the effect of the noise in the training samples. Allowing the system to be integrated into real-world testing systems as well as simulation based systems.

As an additional benefit, KNNR does not require lengthy training phase. This trait could be useful in photonic research and design as the design parameters require precise modifications, which in turn could require the regression system to be re-trained. In such a system, KNNR could drastically improve response time. Additionally, KNNR can be used to increase the resolution of the results of conventionally used numerical methods such as FEM. Once the output of the structure is calculated for some data points, KNNR could be employed to calculate additional data much faster than numerical methods.

It is also possible to use KNNR in estimating the other key propagation features such as dispersion, birefringence and many more. These tasks are left to future works to keep the manuscript concise.

## Declarations

### Author contribution statement

Cem Kalyoncu: Performed the machine learning experiments; Analyzed the data; Wrote the paper.

Ahmet Yasli: Designed the PCF based SPR sensor; Performed the PCF-SPR experiments; Analyzed the data; Wrote the paper.

Huseyin Ademgil: Designed the PCF; Analyzed the general data; Wrote the paper.

### Declaration of interests statement

The authors declare no conflict of interest.

### Data availability statement

Data will be made available on request.

### Funding statement

This research did not receive any specific grant from funding agencies in the public, commercial, or non-profit sectors.

### Additional information

No additional information is available for this paper.
